# Geospatial modeling of microcephaly and zika virus spread patterns in Brazil

**DOI:** 10.1371/journal.pone.0222668

**Published:** 2019-09-26

**Authors:** Pedro Amaral, Lucas Resende de Carvalho, Thiago Augusto Hernandes Rocha, Núbia Cristina da Silva, João Ricardo Nickenig Vissoci

**Affiliations:** 1 CEDEPLAR/UFMG, Center for Development and Regional Planning, Federal University of Minas Gerais, Belo Horizonte, Minas Gerais, Brazil; 2 PAHO/WHO, Brasília, Federal District, Brazil; 3 CEPEAD/UFMG, Center of Higher Studies and Research in Administration, Federal University of Minas Gerais, Belo Horizonte, Minas Gerais, Brazil; 4 Duke University, Duke School of Medicine, Department of Surgery, Division of Emergency Medicine, Durham, North Carolina, United States of America; VA-MD College of Veterinary Medicine, UNITED STATES

## Abstract

Microcephaly and Zika Virus infection (ZIKV) were declared Public Health Emergencies of International Concern by the World Health Organization in 2016. Brazil was considered the epicenter of the outbreak. However, the occurrence of both ZIKV and microcephaly in Brazil was not evenly distributed across the country. To better understand this phenomenon, we investigate regional characteristics at the municipal level that can be associated with the incidence of microcephaly, our response variable, and its relationship with ZIKV and other predictors. All epidemiological data in this study was provided by the Ministry of Health official database (DATASUS). Microcephaly was only confirmed after birth and the diagnostic was made regardless of the mother’s ZIKV status. Using exploratory spatial data analysis and spatial autoregressive Tobit models, our results show that microcephaly incidence is significantly, at 95% confidence level, related not only to ZIKV, but also to access to primary care, population size, gross national product, mobility and environmental attributes of the municipalities. There is also a significant spatial autocorrelation of the dependent variable. The results indicate that municipalities that show a high incidence of microcephaly tend to be clustered in space and that incidence of microcephaly varies considerably across regions when correlated only with ZIKV, i.e. that ZIKV alone cannot explain the differences in microcephaly across regions and their correlation is mediated by regional attributes.

## Introduction

The Zika virus infection (ZIKV) was declared a health emergency by the World Health Organization in 2016 [1]. In 2017, nearly all countries in the tropics were considered autochthonous regions of Zika virus transmission [2]. Brazil was considered the epicenter of the epidemic, with reports of 130,000 ZIKV in 2016 [3].

The fast increase in the number of reports prompted international attention and dedicated research increasingly suggested an association between ZIKV and neurological syndromes such as Guillain-Barré and microcephaly. In 2015 and 2016, 2,229 cases of microcephaly in infants were confirmed in Brazil, compared to a yearly average of only 157 cases between 2000 and 2014 [3].

Several studies of different backgrounds established the link between the ZIKV and microcephaly and other neurological disorders [4–9]. However, the occurrence of both ZIKV and microcephaly in Brazil was not evenly distributed across the country. As shown by Vissoci [10], the geospatial distribution of ZIKV in Brazil was diffuse, with scattered groups of municipalities with high incidence in the Midwest, North, Northeast and Southeast regions. On the other hand, patterns of microcephaly geospatial distribution, distinct from that of ZIKV, tended to be concentrated in the Northeast. Hotspots for microcephaly incidence varied less between the regions across all investigated bi-monthly periods in comparison with the varied locations of ZIKV incidence hotspots [10]. This spatial disparity in the co-occurrence of ZIKV and microcephaly suggests that there are other factors mediating the relationship between them. Hence, in this paper our objective is to investigate whether regional attributes such as deprivation, sanitation, mobility, environment and other socioeconomic characteristics of the municipalities play any significant role in the correlation between microcephaly and ZIKV. By using a framework of health geography, we can provide insights into disease spread patterns, high-risk areas, and correlated regional attributes that allow for inferences regarding the determinants of these outcomes [11].

The use of geospatial modeling techniques has proven its value for studying infections such as dengue fever [12] and chikungunya [13]. Health geography studies have been used to identify high-risk areas, considering the presence of elements capable to predict the levels associated with the incidence of arbovirus diseases. The geospatial approach is able to create insights to support health policies and surveillance strategies dedicated to minimizing the negative consequences of ZIKV. Despite this potential, few studies have examined the spatial patterns associated with ZIKV and the neurological conditions resulting from the infection [10].

The regional imbalances in the incidence of both microcephaly and ZIKV suggest that regional attributes have also played an important role in the spread of Zika virus and in mediating its association with microcephaly. Jaenisch et al. [14] found the estimated risk that a baby born to a woman infected by the Zika virus during pregnancy would have microcephaly varied substantially across Brazil. According to them, geographical area is one of the main factors affecting the risk. However, the investigation of geographical attributes related to the incidence of microcephaly was not one of the objectives of their study, an issue we address with this research.

Taking into consideration the lack of studies that evaluate the regional incidence of microcephaly and ZIKV, here we investigate the regional characteristics at the municipal level that can be associated with the incidence of microcephaly and the relation between the disease and ZIKV infection.

## Methods

We designed an ex-post-facto ecological study based on routinely collected health data following the RECORD protocol [15]. Given the use of variables referring to past events that were not specifically collected for this study, there was no control by the researchers regarding the form of collection. The Brazilian Universal Health System (SUS) [16] is responsible for sharing all information collected regarding the epidemiological situation in the country. All epidemiological data used in this work was gathered from DATASUS, which is the department of the Ministry of Health (MoH) responsible for publicizing health system databases. The data was aggregated to the level of the Brazilian municipalities. Figures on confirmed ZIKV cases were obtained from the Disease Notification Information System [3]. A case is considered as confirmed if one of the following circumstances are met: viral RNA track, positive viral detection or IgM serology. The information on newborns with confirmed microcephaly was obtained from the System for Specialized Management Support [17]. To be considered as confirmed microcephaly case the following criteria should be met: infant with 37 or more weeks of gestation with a head circumference equal to or less than 31.9 cm for male infants, or equal to or less than 31.5 cm for female infants, in concurrence with WHO standards [1]. Microcephaly was only confirmed after birth and the diagnostic was made regardless of the ZIKV status of the mother. The volume of cases related to ZIKV and microcephaly were weighted by the population at the municipality level. The municipality’s population size was obtained from the Brazilian Institute of Geography and Statistics [18] repository and refers to all inhabitants living in each municipality in 2016. Data related to primary care coverage was obtained from the MoH. All data covering ZIKV and microcephaly was categorized by quarters in the period from January to December, 2016.

In addition to the data listed above, we used information on the urban structure of the Brazilian municipalities. This data was obtained from the Brazilian index of urban structure (IBEU) and refers to 2013. The IBEU is based on several dimensions associated with sanitation, urban structure and health conditions. Data on the IBEU was provided by the Observatory of the Metropoles of the National Institute of Science and Technology [19].

The IBEU covers five urban dimensions: mobility, environmental conditions, housing conditions, sanitation and infrastructure. The mobility dimension assesses the proportion of inhabitants who commute between home and work for at least one hour. The environmental aspect evaluates the lack of rubbish around residences, the existence of open sky sewage and afforestation index. Housing conditions are a composite score related to five indicators: proportion of people living in shanty towns, number of bedrooms with a maximum of two people, number of households with a maximum ratio of 4 people per restroom, proportion of households whose walls are made of bricks or appropriate wood and proportion of inadequate households. The sanitation dimension covers aspects related to four indicators: households with adequate sewage, homes with appropriate water and sewage services, coverage by garbage collection service, and availability of energy services. The last dimension regarding the urban structure comprises the infrastructure index with the following metrics: the proportion of people living in households covered by public illumination, streets made of asphalt or concrete paving, and household identification. The IBEU was calculated considering all indicators with the same weight and computing an average score covering all five dimensions. The final IBEU varied from 0 to 1. Finally, to better characterize the socioeconomic status of each municipality, we consider information on the Gross Domestic Product (GDP) per capita in 2013, provided by Brasil-IBGE [20].

The variable of interest in this study is the incidence of microcephaly in each municipality in Brazil. However, of the 5,560 municipalities in the sample, 4,831 (86.9%) did not register any incidence of microcephaly in the fourth quarter of 2016. Incidence rate mapping and the Getis-Ord-G_*i*_ cluster analysis [21] were performed in ARCGIS 10.3 [22]. Given distribution of the data, to estimate the correlation between regional attributes and incidence of microcephaly we used a Tobit model approach.

Censored regression models or Tobit models can be applied when the variable of interest is censored, i.e., its values are not observable beyond a given limit. The Tobit model, developed by James Tobin [23], estimates the relationship between a non-negative variable and independent variables. The generic specification of a Tobit model is described as follows:

$$y_{i}^{⋆}=x_{\;i}\beta +\varepsilon_{\;i}$$ (1)

$$y_{i\;}=0\;if\;y_{i}^{⋆}\leq 0$$

$$y_{\;i}=y_{i}^{⋆}\;if\;y_{i}^{⋆}>\;0$$

where y* indicates a latent variable that depends on the matrix of explanatory variables (*x*) and a vector of parameters *β* and a normally distributed error term *ε*.

The choice of the Tobit estimator is justified by the statistical model that can generate the type of data used in this analysis. As stated by Amemiya [24], we cannot use any continuous density because a continuous density is inconsistent with the fact that there are several observations at 0, as is the case of the distribution of incidence of microcephaly. However, the observed values of incidence of microcephaly are continuous beyond zero and have no upper bound. Hence, we also rule out binary or count models. Another important characteristic of our model is that the exogenous variables are observable regardless of the value of y*, so the model can be labeled as a censored regression model [24].

The estimator presented in Eq (1) does not account for the presence of spatial correlation, which may render the Tobit model estimation inefficient or even inconsistent [25]. Given the spatial pattern of both microcephaly and ZIKV, this is a hypothesis that we must investigate. In order to incorporate spatial correlation, a SAR Tobit (spatial autoregressive Tobit) was estimated as presented in Eq 2.

$$y_{i}^{⋆}=S^{-1}x_{\;i}\beta +S^{-1}\varepsilon_{\;i}$$ (2)

$$y_{i}=0\;if\;y_{i}^{⋆}\;\leq 0$$

$$y_{\;i}=y_{i}^{⋆}\;if\;y_{i}^{⋆}>\;0$$

$$S=I_{n}-\rho W$$

where I_n_ indicates an identity matrix of order n, ρ is a spatial autoregressive parameter and W is a spatial weights matrix, assumed here as first-order Queen contiguity type.

Although these models have been implemented through maximum-likelihood (MLE), their estimation demands high computational costs. The Bayesian approach provides a less demanding computational alternative. In particular, a Monte Carlo Bayesian approach was applied based on the Markov Chain (MCMC) and the Gibbs algorithm. The implementation used for the SAR Tobit model was proposed by [26] and implemented in R by the *spatialprobit* package [27].

The potential presence of a significant spatial lag parameter implies that variations in the exogenous variables have not only direct effects on the dependent variable, but also indirect effects due to the spatial spillover. Therefore, the total effect of an explanatory variable *x* on the dependent variable *y* can be decomposed into direct effects and indirect effects. The direct effects refer to the predicted direct impact of variations in *x* on the variable of interest *y* in the same municipality. The indirect effects are the impacts due to spatial spillovers of the variations of *y* within the neighboring municipalities. The compound effect of variations of *x* on *y* are, therefore, the sum of both direct and indirect effects.

## Results

**Fig 1:**
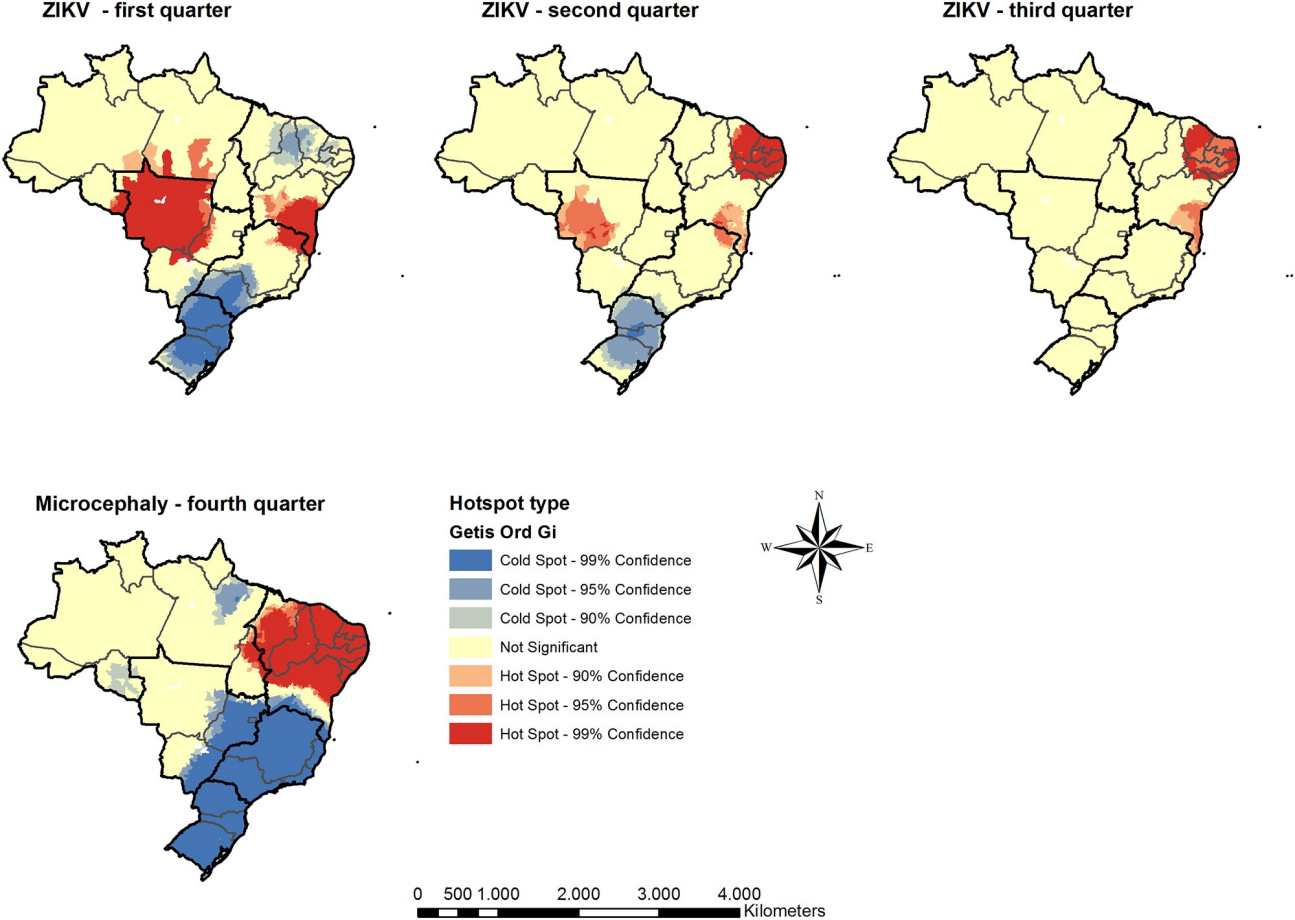
Spatial autocorrelation measured by Getis-Ord Gi of ZIKV and microcephaly, Brazil– 2016. Source: Own elaboration with SINAN, 2016 database.

Fig 1 shows the spatial autocorrelation of the main variables in this study, i.e. microcephaly incidence in the fourth quarter of 2016 and ZIKV in the first three quarters. In this study, the most important aspect of the spatial distribution of microcephaly and ZIKV is their spatial concentration, which indicates a possible spillover effect. The coincidence of areas, especially between ZIKV the second quarter of the year and microcephaly in the last quarter is also an important aspect to highlight which is further explored in this study.

The results of the estimation of the spatial Tobit models are shown in the tables that follow. Given the presence of a significant spatial lag coefficient, the Tobit parameters cannot be directly interpreted as in a regular Tobit, due to the spatial multiplier effect. To allow for marginal interpretations, the main results are followed by direct, indirect and total effects on the dependent variable of marginal changes in the explanatory variables.

Tables 1 and 2 show the results of the regression of microcephaly incidence only on ZIKV incidence. These results are shown to provide comparability against those in Tables 3 and 4, which add regional attributes as explanatory variables. This way, we can evaluate how regional attributes mediate the relationship between ZIKV and microcephaly. Given that our model is based on Bayesian MCMC estimates, the significance inference was based on p-levels instead of standard t-statistics. For more information on inference based on Bayesian MCMC estimates, see [28].

**Table 1:** Results of SAR Tobit—Dependent variable: Microcephaly incidence (4^th^ quarter), Brazil—2016.

Variable	Posterior mean	Standard deviation	p-level	
Constant	-20.4983	1.2941	0.000	☨
ZIKV incidence (3^rd^ quarter)	-0.0650	0.0393	0.044	☨
ZIKV incidence (2^nd^ quarter)	0.0845	0.0475	0.037	☨
ZIKV incidence (1^st^ quarter)	-0.0197	0.0113	0.040	☨
Sig_e	562.01	59.82	0.000	☨
Rho	0.3345	0.0381	0.000	☨

^☨^Significant at the 95% confidence level.

Source: Own elaboration.

**Table 2:** Results of SAR Tobit—Direct and indirect effects—Dependent variable: Microcephaly incidence (4^th^ quarter), Brazil—2016.

Variable	Lower 0.05	Posterior mean		Upper 0.95
(a) Direct effects				
ZIKV incidence (3^rd^ quarter)	-0.0648	-0.0317	☨	-0.001
ZIKV incidence (2^nd^ quarter)	0.0035	0.0413	☨	0.071
ZIKV incidence (1^st^ quarter)	-0.0187	-0.0097	☨	0.002
(b) Indirect effects				
ZIKV incidence (3^rd^ quarter)	-0.1836	-0.0826	☨	-0.004
ZIKV incidence (2^nd^ quarter)	0.0039	0.0979	☨	0.235
ZIKV incidence (1^st^ quarter)	-0.0363	-0.0151	☨	-0.002
(c) Total effects				
ZIKV incidence (3^rd^ quarter)	-0.2545	-0.1242	☨	-0.005
ZIKV incidence (2^nd^ quarter)	0.0137	0.1616	☨	0.315
ZIKV incidence (1^st^ quarter)	-0.0735	-0.0378	☨	-0.003

^☨^Significant at the 95% confidence level.

Source: Own elaboration.

**Table 3:** Results of SAR Tobit—Dependent variable: Microcephaly incidence (4^th^ quarter), Brazil—2016.

Variable	Posterior mean	Standard deviation	p-level	
Constant	-91.9230	9.9291	0.000	☨
ZIKV incidence (3^rd^ quarter)	-0.0612	0.0431	0.043	☨
ZIKV incidence (2^nd^ quarter)	0.0716	0.0476	0.040	☨
ZIKV incidence (1^st^ quarter)	-0.0100	0.0084	0.114	
Primary care	0.1218	0.0270	0.000	☨
Ln municipal GDP	-20.0970	1.8606	0.000	☨
Ln population	39.2147	2.6992	0.000	☨
Mobility	18.4701	6.8232	0.004	☨
Environmental	-11.5750	3.6015	0.000	☨
Housing	-7.0651	8.1223	0.186	
Sanitation	-4.6374	3.4631	0.090	
Infrastructure	6.9194	4.7694	0.082	
Sig_e	336.9353	27.5831	0.000	☨
Rho	0.2401	0.0378	0.000	☨

^☨^Significant at the 95% confidence level.

Source: Own elaboration.

**Table 4:** Results of SAR Tobit—Direct and indirect effects—Dependent variable: Microcephaly incidence (4^th^ quarter), Brazil—2016.

Variable	Lower 0.05	Posterior mean		Upper 0.95
*(a) Direct effects*				
ZIKV incidence (3^rd^ quarter)	-0.0532	-0.0254	☨	0.0000
ZIKV incidence (2^nd^ quarter)	0.0006	0.0308	☨	0.0620
ZIKV incidence (1^st^ quarter)	-0.0125	-0.0056		0.0020
Primary care	0.0394	0.0580	☨	0.0770
Ln municipal GDP	-10.9581	-9.4500	☨	-7.9490
Ln population	16.2770	18.4727	☨	20.6540
Mobility	3.6007	8.9145	☨	14.1640
Environmental	-8.3755	-5.5626	☨	-2.6920
Housing	-10.0473	-3.6000		2.5940
Sanitation	-5.2690	-2.3451		0.5640
Infrastructure	0.0107	3.5152		6.8470
*(b) Indirect effects*				
ZIKV incidence (3^rd^ quarter)	-0.1565	-0.0750	☨	0.0000
ZIKV incidence (2^nd^ quarter)	0.0018	0.0912	☨	0.1840
ZIKV incidence (1^st^ quarter)	-0.0370	-0.0166		0.0050
Primary care	0.1164	0.1715	☨	0.2270
Ln municipal GDP	-32.3536	-27.9277	☨	-23.5740
Ln population	48.2199	54.5917	☨	60.8530
Mobility	10.5738	26.3474	☨	41.8960
Environmental	-24.6091	-16.4406	☨	-7.9400
Housing	-29.5589	-10.6396		7.6620
Sanitation	-15.5699	-6.9310		1.6560
Infrastructure	0.0313	10.3907		20.2350
*(c) Total effects*				
ZIKV incidence (3^rd^ quarter)	-0.2099	-0.1003	☨	0.0000
ZIKV incidence (2^nd^ quarter)	0.0024	0.1220	☨	0.2460
ZIKV incidence (1^st^ quarter)	-0.0495	-0.0222		0.0060
Primary care	0.1558	0.2295	☨	0.3040
Ln municipal GDP	-43.2407	-37.3777	☨	-31.5110
Ln population	64.5281	73.0644	☨	81.4700
Mobility	14.1721	35.2618	☨	56.0620
Environmental	-32.9348	-22.0033	☨	-10.6250
Housing	-39.6063	-14.2396		10.2550
Sanitation	-20.8298	-9.2761		2.2200
Infrastructure	0.0420	13.9060		27.0660

^☨^Significant at the 95% confidence level.

Source: Own elaboration.

The results presented in Tables 3 and 4 show that microcephaly incidence in Brazil is significantly and positively related to access to primary care, population size and mobility index of the municipalities. On the other hand, microcephaly incidence shows a negative significant association with GDP and environmental index of the municipalities.

There is also a significant positive spatial autocorrelation of the dependent variable. This spatial autocorrelation is measured by the coefficient *ρ*, which estimated mean is of 0.24. This value is more than 6 standard deviations away from zero, indicating that municipalities that have a high incidence of microcephaly tend to be significantly spatially clustered, even after controlling for their attributes and ZIKV incidence. To evaluate the evolution of the relationship between microcephaly and ZIKV across different time lapses between notifications, the incidence of ZIKV was considered separately for the first three quarters of 2016, while microcephaly incidence was considered for the fourth quarter only. The results show a significant positive relationship between ZIKV incidence in the second quarter of the year and microcephaly, a significant negative relationship between ZIKV incidence in the third quarter of the year and microcephaly, and a non-significant relationship between microcephaly and ZIKV in the first quarter.

The effect of each of the significant explanatory variables on the incidence of microcephaly can be seen in Table 4. Municipal GDP (logged) exerts a total effect of -37.4. In other words, for every 1% increase in the GDP, the incidence of microcephaly decreases by 0.37 points, being -0.095 points in direct effects and -0.279 in indirect effects. Primary care coverage exerts a compound effect of 0.229, i.e. for each additional percentage point of coverage, microcephaly incidence is raised 0.23 points, being 0.06 in direct effects and the remaining 0.17 in indirect effects. Large municipalities tend to present higher microcephaly incidence, so that a 1% increase in population size is related to a 0.73 increase in microcephaly, being 0.18 direct and 0.55 indirect effects. For the IBEU indices, mobility has a total impact of 35 points and environment of -22. Since these indices vary between 0 and 1, we have that an increase of 0.01 point in the indices is related to 0.35 increase in microcephaly for the mobility index and 0.22 decrease in microcephaly for the environmental index.

The total effect of ZIKV on microcephaly is of -0.10 for the third quarter and 0.12 for the second quarter. These mean results are different from those shown for the model considering only ZIKV as an explanatory variable and no measure of regional attributes. When no regional variable was considered, the results were -0.12 and 0.16, respectively. Hence, not controlling the relationship between ZIKV and microcephaly for different regional attributes may overestimate the correlation between ZIKV and microcephaly.

## Discussion

Our results show that regional attributes can significantly contribute to explaining microcephaly incidence rates across Brazilian municipalities and that disregarding these attributes may lead to overestimation of the magnitude of the relationship between ZIKV and microcephaly. Municipalities with greater GDP per capita and with better environmental urban structures show a lower incidence of microcephaly. The effect of greater GDP per capita may be related to a smaller malnutrition rate and better knowledge of the effects of drug abuse during gestation, which are potential causes of microcephaly [29]. On its turn, a better environmental structure may lead to lower reproductive rates of mosquitoes, especially *Aedes aegypti*, potentially affecting the probability of infection of other viruses such as dengue or chikungunya, which may also cause microcephaly.

On the other hand, municipalities with larger population size and better mobility index show a greater incidence of microcephaly. This may be due to the easier spread of other infections that can also result in microcephaly, such as rubella, cytomegalovirus, toxoplasmosis meningitis and HIV. Primary care coverage was also found to be positively correlated with microcephaly incidence. This correlation may be linked to better diagnosis and information on microcephaly.

Regarding the relationship between ZIKV and microcephaly, the positive association between the notification of ZIKV in the second quarter of 2016 and confirmation of microcephaly in the fourth quarter is expected from previous literature given the higher risk of microcephaly when ZIKV infection occurs in the first trimester of pregnancy. However, the negative relationship between ZIKV in the third quarter of the year and microcephaly in the fourth quarter is intriguing. One possible explanation is a fast reduction of ZIKV in municipalities with high levels in the previous term. Given the time lapse between ZIKV and microcephaly notifications, municipalities with a significant drop of ZIKV in the third quarter should show a lower incidence of microcephaly only in the first quarter of the following year. As shown in Fig 1, the incidence rate of ZIKV changed very fast in Brazil, especially in the Western region of the country, which may be a conjoint result of public policies to reduce the population of *A*. *aegypti* and a decrease in average temperatures in the third quarter.

In sum, ZIKV incidence is very important to predict microcephaly incidence. However, regional attributes also play a significant role in explaining the differences in microcephaly incidence and, at least partially, may be cofactors that explain the varied risk ratio of microcephaly in pregnant women infected with ZIKV found by Jaenisch et al. [14].

## Final remarks

The emergence of ZIKV and microcephaly was considered of global interest because of the quick increase in the number of countries facing an active circulation of ZIKV and the evidence linking this disease with microcephaly. Adequate monitoring of the spread patterns of ZIKV and incidence of microcephaly are essential to support actions aiming at an adequate response to this epidemiological crisis. Taking into consideration this challenge, the main objective of this work was to evaluate how past ZIKV incidence and regional cofactors can help to understand the spatial spread pattern of microcephaly.

The capability to anticipate future regions that might face a high volume of microcephaly cases resulting from a previous peak in ZIKV incidence can support the ability to provide appropriate emergency response interventions. Aiming at the minimization of the negative consequences of ZIKV and microcephaly, public policies can be better implemented once the right locations can be prioritized based on risk levels. The model developed in this study can act as a tool to support the formulation of responses to ZIKV and microcephaly health crisis in this new frontier.
